# Internet Survey of Risk Factors Associated With Training and Competition in Dogs Competing in Agility Competitions

**DOI:** 10.3389/fvets.2021.791617

**Published:** 2022-01-04

**Authors:** Arielle Pechette Markley, Abigail B. Shoben, Nina R. Kieves

**Affiliations:** ^1^The Ohio State University Veterinary Medical Center, Columbus, OH, United States; ^2^Division of Biostatistics, The Ohio State University College of Public Health, Columbus, OH, United States; ^3^Department of Veterinary Clinical Sciences, The Ohio State University College of Veterinary Medicine, Columbus, OH, United States

**Keywords:** agility, canine, sports medicine, injury, training, competition

## Abstract

**Objective:** To describe risk factors associated with training and competition in relation to frequency and severity of injuries experienced by agility dogs.

**Procedures:** An internet-based survey collected data on competition level variables and training level variables. The primary outcome was history of any injury and a secondary outcome considered history of severe injury (injury lasting > 3 months). Logistic regression was used to estimate associations and final models were obtained *via* backward selection to identify the strongest associations within variables.

**Results:** There were 4,197 dogs included in this analysis. Injury was reported for 1,737 (41.4%) dogs and severe injury was reported for 629 (15.0%). In the model with competition level factors, jumping 4” (OR: 1.50) or 2–4” (OR: 1.31) over shoulder height compared to jumping 0–2” lower and competing at national events was associated with increased injury risk, while competing 6+ times on rubber matting was associated with lower risk (OR: 0.62). Training level variables associated with injury risk were age starting jump, teeter, and weave training, with the highest risk observed for dogs starting jump training between 3 and 18 months but starting weave and teeter training after 18 months of age.

**Conclusion and Clinical Relevance:** Many variables thought to be associated with injury risk were not significant in the final model. Starting jump training at an earlier age was associated with greater risk of injury relative to starting after 18 months. It is possible that the high impact of jump training before skeletal maturity may increase the risk of injuries or musculoskeletal conditions. The increased risk of injury in dogs that jump 2–4, or 4+ inches higher than shoulder height may be due to increased biomechanical forces during takeoff and landing. Faster dogs may be at higher risk of injury; handlers planning competition around big events or competing at the national level are likely to have faster dogs, and may be less likely to compete on rubber matting. These data provide valuable current insight into the possible effects that training and competition variables may have on injury risk in agility dogs.

## Introduction

Dog agility is a popular performance event that has grown rapidly in the past decade. Entries for American Kennel Club agility events have increased by 38% over the last decade.^1^ As the popularity of agility has increased, reported injury rates have also increased from 32% in 2009 to 41.7% in 2019 (1). The cause of the increased injury rate is likely multifactorial (1). Risk factors associated specifically with training and competition have been minimally evaluated. A previous study, performed by Cullen et al., used multivariable techniques to evaluate some potential risk factors for agility injuries (2). Data collected in this study only found the use of alternative therapeutic treatments to be associated with higher odds of injury (2).

In racehorses, the training and competition level risk factors for catastrophic musculoskeletal injury have been thoroughly investigated. Factors such as age at first start, higher race class, surface condition (firmer turf or wetter conditions on dirt), longer race distance, greater number of starts, longer career length, and previous injury have all been consistently shown to increase risk of catastrophic musculoskeletal injury in racehorses (3). The literature evaluating competition and training risk factors in human athletics is extensive and risk factors vary significantly by sport (4–10). Many similar training and competition variables exist in canine agility but have not been previously evaluated in relation to injury risk. At this time, there is no published data regarding the effect of variables such as jump height, level of competition, age at which training and competition was started, surface condition, and a variety of other factors that may play a role in increasing or decreasing injury risk.

While the obstacles that comprise agility courses have, for the most part, stayed the same over the past decade, the technicality of course design has increased (11, 12). This has resulted in changes to both handling and training techniques. There are a variety of ways of training each of the obstacles and as course speeds have increased there are trends in training obstacles to increase speed. However, risk of injury associated with types of training techniques or age at time of training certain techniques has not been evaluated.

The aim of this study was to thoroughly evaluate variables in canine agility training and competition that may affect the prevalence and severity of injuries agility dogs sustain. We wanted to specifically evaluate training-specific and competition-level factors that might be associated with injury history and describe the association between them and injury history. We hypothesized that early jump training, jumping higher jump heights, stopped contact training, and increasing number of trial weekends per year or runs per day would be associated with increased injury risk. We also hypothesized that planned time off would be associated with decreased injury risk.

## Materials and Methods

The internet-based survey utilized has been described previously (1). In brief, the survey was conducted in English and was distributed primarily *via* social media during a 6-week period in the fall of 2019 with University Institutional Review Board approval.^2^,^3^,^4^ Eligible dogs had competed in at least one agility competition in the preceding 3 years. We classified our major variables of interest as “demographic variables” (both handler and dog), “competition variables” (e.g., primary competition venue, competition surface), and “training variables” (e.g., age at which agility training started, and methods for training different obstacles).

Competition level variables examined were: jumping height difference (jump height – height at the withers), primary organization, highest level achieved, number of trial weekends per year, number of days competing per trial weekend, number of runs per day, number of times the dog had competed at the national level, and number of times the dog had competed at the international level. Also examined was the frequency of competing on various surfaces. Surfaces included grass, sand, dirt, turf, rubber, foam, and other. Handlers were asked how they planned their training and competition schedules – around a big event, around availability of trials/schedule, a mix of the two, or other.

Training level variables were reported age starting any agility training, age starting each specific agility obstacle, age competing in first trial, age competing in first fun match, the behavior the dog performs at the end of each contact obstacle, and the method for training the weave obstacle. Contact obstacles are defined as having a “contact zone” where the dog must touch any part of one foot prior to exiting the obstacle for the A-frame and dogwalk, and where the dog must touch the “up” contact zone when ascending and then “down” contact zone one the plank touches the ground for the teeter obstacle. For the purpose of this study the contact obstacles evaluated for training techniques included the A-frame, teeter, dogwalk and weave obstacles. The training techniques evaluated for the A-frame and dogwalk were: (1) 2-on 2-off, defined as stopping with the front two feet on the ground and the rear two feet in the contact zone of the obstacle; (2) All 4 on, defined as stopping with all 4 paws in the contact zone; (3) Running, defined as moving up and over the obstacle without stopping; and (4) Other. The training techniques evaluated for the teeter were: (1) 2-on 2-off; (2) All 4 on in a down position; (3) All 4 on in a standing position; (4) Other specific trained behavior; and (5) No specific trained behavior. Weave obstacle training techniques included: (1) 2 ×2, defined as starting with a single set of 2 weave poles and systematically adding 2 poles; (2) Channel method, defined as where the weave poles are offset so that a “channel” is formed between the two lines of poles and eventually the channel is closed so that the dog learns the weaving motion; (3) Guide wires, defined as where the weave poles are set at competition standards, but guide wires are attached so that the dog is funneled between the poles so that the dog must continue straight; (4) Other.

Our outcome of interest was injury history, defined as an injury that kept the dog from participating in agility for over a week. A secondary outcome of “severe” injury was defined as at least one injury that kept the dog from participating in agility for <3 months (4 months or longer), or lead to retirement from agility.

All models were adjusted for dog age to account for greater lifetime injury risk among older dogs. Associations between each competition and training variables with injury history were first assessed in dog age adjusted logistic regression models. Final adjusted models were constructed separately for competition and training variables to explore adjusted associations. All models were built using backward selection, starting with candidate variables associated with the outcome at *p* < 0.20 in dog age only adjusted models and ending with all variables associated with the outcome at *p* < 0.05. Using backward selection, the variable with the largest *p*-value was removed at each step until all remaining variables were associated with the outcome at *p* < 0.05. We used an available case approach to missing data, after restricting to those who completed >90% of the survey and answered our primary outcome question. This process was repeated for the outcome of severe injury. All analyses were conducted using Stata.

## Results

Our sample included 4,197 responses that had >90% survey completion and provided an answer to the primary injury history question. Of the 4,197 dogs, 1,737 (41.4%) reported an injury history and 629 (15.0%) reported a history of severe injury.

Nearly all competition level factors were associated with injury history in age-adjusted models (Table 1). After backward selection, the difference between jump height and dog height, average runs per trial day, times competing at the national level, planning around big events, and competing on rubber matting were associated with injury risk at *p* < 0.05 (Table 2). In this model, dogs jumping 4 or more inches above their height had the highest risk of injury (OR: 1.50 compared to dogs jumping 0–2” below their height), and dogs jumping 2–4” above their height were also at increased risk (OR: 1.31). The lowest risk was observed for dogs jumping 2–4” below their height. Dogs completing 3–4 runs per trial day had a greater risk of injury history (OR: 1.15) than dogs with only 1–2 runs per day, but dogs completing 5 or more runs per trial day had a lower risk than both groups (OR: 0.92 compared to the 1–2 runs per day group).

**Table 1:** Age adjusted associations between competition risk factors and injury history.

	***N* (%)**	**Any injury OR (95% CI)**	**Any injury *p*-value**	**Severe injury** **OR (95% CI)**	**Severe injury *p*-value**
Primary organization			<0.001^a^		0.56
AKC	1,172 (27.9)	REFERENCE		REFERENCE	
CPE	344 (8.2)	0.79 (0.61, 1.01)		0.89 (0.64, 1.26)	
USDAA	296 (7.1)	1.37 (1.05, 1.78)		1.11 (0.79, 1.58)	
NADAC	112 (2.7)	0.88 (0.58, 1.33)		1.10 (0.65, 1.88)	
AAC (Canada)	225 (5.4)	1.06 (0.79, 1.42)		0.69 (0.44, 1.08)	
Other North American	813 (19.4)	0.84 (0.70, 1.02)		0.99 (0.76, 1.27)	
FCI agility	756 (18.0)	1.40 (1.16, 1.69)		0.96 (0.74, 1.26)	
Other non-North American	477 (11.4)	1.26 (1.01, 1.58)		1.17 (0.87, 1.58)	
Highest level achieved			0.013^a^		0.39
Entry level	596 (14.2)	0.74 (0.60, 0.91)		0.82 (0.60, 1.13)	
Intermediate level	766 (18.3)	1.00 (0.84, 1.19)		1.05 (0.83, 1.33)	
High level	2,829 (67.5)	REFERENCE		REFERENCE	
Jump height difference			<0.001^a^		0.090^a^
Jumping > 4” above height	144 (3.5)	1.70 (1.19, 2.43)		1.22 (0.76, 1.95)	
Jumping 2–4” above height	299 (7.3)	1.47 (1.13, 1.91)		1.11 (0.77, 1.59)	
Jumping 0–2” above height	853 (20.9)	1.05 (0.87, 1.26)		0.91 (0.70, 1.18)	
Jumping 0–2” below height	1,158 (28.4)	REFERENCE		REFERENCE	
Jumping 2–4” below height	797 (19.6)	0.74 (0.61, 0.90)		0.72 (0.55, 0.95)	
Jumping 4–6” below height	485 (11.9)	0.91 (0.73, 1.14)		0.87 (0.65, 1.17)	
Jumping > 6” below height	339 (8.3)	0.95 (0.74, 1.22)		1.14 (0.83, 1.56)	
Approach to competition planning			<0.001^a^		<0.001^a^
Plan around availability/schedule	2,801 (67.0)	REFERENCE		REFERENCE	
Plan around a big event	101 (2.4)	1.61 (1.07, 2.42)		1.93 (1.18, 3.17)	
Mix of the two	1,107 (26.5)	1.50 (1.30, 1.73)		1.32 (1.08, 1.60)	
Other approach	171 (4.1)	0.89 (0.64, 1.24)		1.36 (0.91, 2.05)	
Advance competition planning			0.078^a^		0.051^a^
1–2 months	1,533 (36.7)	REFERENCE		REFERENCE	
3–6 months	1,910 (45.7)	1.15 (1.00, 1.32)		1.17 (0.96, 1.43)	
6–12 months	631 (15.1)	1.25 (1.03, 1.52)		1.42 (1.10, 1.83)	
12+ months	104 (2.5)	0.97 (0.64, 1.47)		0.94 (0.52, 1.70)	
Trial weekends per year			0.002^a^		0.050^a^
<5 weekends	448 (10.7)	0.79 (0.58, 1.07)		1.82 (1.16, 2.86)	
5–10 weekends	918 (21.9)	0.95 (0.73, 1.25)		1.54 (1.02, 2.34)	
11–15 weekends	1,082 (25.8)	1.12 (0.86, 1.46)		1.62 (1.08, 2.43)	
16–20 weekends	906 (21.6)	1.25 (0.95, 1.64)		1.92 (1.28, 2.89)	
21–25 weekends	530 (12.7)	1.18 (0.88, 1.58)		1.58 (1.02, 2.46)	
26+ weekends	304 (7.3)	REFERENCE		REFERENCE	
Average runs per trial day			0.005^a^		0.088^a^
1–2 runs per day	1,067 (25.5)	REFERENCE		REFERENCE	
3–4 runs per day	2,444 (58.4)	1.25 (1.07, 1.45)		1.23 (1.00, 1.52)	
5+ runs per day	677 (16.2)	1.02 (0.83, 1.25)		1.02 (0.77, 1.35)	
Average days per trial weekend			0.32		0.10^a^
Only 1 day	485 (11.6)	1.10 (0.89, 1.36)		1.16 (0.87, 1.54)	
1 or 2 days; it depends	1,680 (40.1)	1.03 (0.89, 1.18)		1.10 (0.91, 1.33)	
Usually 2 days, sometimes 3	1,701 (40.6)	REFERENCE		REFERENCE	
As many as possible (often 3)	320 (7.6)	0.84 (0.65, 1.08)		0.71 (0.49, 1.03)	
Grass surface			<0.001^a^		0.25
Never competed	767 (18.3)	REFERENCE		REFERENCE	
<6 times per year	1,882 (45.0)	1.32 (1.10, 1.58)		1.21 (0.93, 1.57)	
6+ times per year	1,536 (36.7)	1.50 (1.25, 1.81)		1.25 (0.96, 1.63)	
Dirt surface			0.46		0.90
Never competed	1,597 (38.2)	REFERENCE		REFERENCE	
<6 times per year	1,698 (40.6)	0.98 (0.84, 1.13)		1.02 (0.83, 1.24)	
6+ times per year	890 (21.3)	1.08 (0.91, 1.28)		0.96 (0.76, 1.22)	
Sand surface			0.042^a^		0.74
Never competed	2,645 (63.2)	REFERENCE		REFERENCE	
<6 times per year	1,253 (29.9)	1.19 (1.04, 1.37)		0.96 (0.80, 1.17)	
6+ times per year	287 (6.9)	1.09 (0.85, 1.40)		0.87 (0.60, 1.26)	
Turf surface			0.85		0.11^a^
Never competed	1,660 (39.7)	REFERENCE		REFERENCE	
<6 times per year	1,095 (26.2)	0.95 (0.81, 1.12)		1.26 (1.02, 1.57)	
6+ times per year	1,430 (34.2)	0.99 (0.85, 1.14)		1.13 (0.92, 1.39)	
Foam surface			0.43		0.70
Never competed	3,522 (84.2)	REFERENCE		REFERENCE	
<6 times per year	494 (11.8)	0.88 (0.72, 1.07)		0.89 (0.68, 1.17)	
6+ times per year	169 (4.0)	0.96 (0.69, 1.33)		0.95 (0.61, 1.47)	
Rubber surface			0.001^a^		0.020^a^
Never competed	2,761 (66.0)	REFERENCE		REFERENCE	
<6 times per year	1,054 (25.2)	0.95 (0.82, 1.10)		1.03 (0.84, 1.25)	
6+ times per year	370 (8.8)	0.64 (0.51, 0.81)		0.62 (0.44, 0.88)	
Other surface			0.15^a^		0.41
Never competed	3,972 (94.9)	REFERENCE		REFERENCE	
<6 times per year	141 (3.4)	1.36 (0.97, 1.92)		0.78 (0.47, 1.31)	
6+ times per year	72 (1.7)	1.25 (0.77, 2.01)		0.67 (0.31, 1.48)	
Times competed at National level			<0.001^a^		0.22
0 (never)	2,539 (61.0)	REFERENCE		REFERENCE	
1	532 (12.8)	1.21 (1.00, 1.47)		1.23 (0.94, 1.59)	
2	352 (8.5)	1.66 (1.32, 2.09)		1.28 (0.95, 1.72)	
3	195 (4.7)	1.40 (1.04, 1.89)		1.03 (0.69, 1.54)	
4	124 (3.0)	2.15 (1.47, 3.14)		1.66 (1.07, 2.57)	
5	76 (1.8)	1.18 (0.74, 1.88)		1.21 (0.68, 2.15)	
>5	345 (8.3)	1.51 (1.19, 1.91)		1.07 (0.79, 1.46)	
Times competed at International level			0.004^a^		0.95
0 (never)	3,909 (93.4)	REFERENCE		REFERENCE	
1	83 (2.0)	1.85 (1.19, 2.89)		0.94 (0.49, 1.80)	
>1	192 (4.6)	1.36 (1.01, 1.83)		0.95 (0.63, 1.43)	

a *p < 0.20 and retained for model building*.

**Table 2:** Coefficients from adjusted model with competition level factors only for any injury.

	**Adjusted OR (95% CI)**	**Adjusted** ***p*-value**
Dog age (per 1 year older)	1.15 (1.12, 1.17)	<0.001
Jump height difference		0.003
Jumping > 4” above height	1.50 (1.04, 2.16)	
Jumping 2–4” above height	1.31 (1.00, 1.71)	
Jumping 0–2” above height	1.01 (0.83, 1.21)	
Jumping 0–2” below height	REFERENCE	
Jumping 2–4” below height	0.78 (0.64, 0.95)	
Jumping 4–6” below height	0.98 (0.78, 1.24)	
Jumping >6” below height	1.10 (0.85, 1.43)	
Approach to competition planning		0.002
Plan around availability/schedule	REFERENCE	
Plan around a big event	1.30 (0.85, 1.99)	
Mix of the two	1.34 (1.15, 1.57)	
Other approach	0.91 (0.64, 1.27)	
Average runs per trial day		0.031
1–2 runs per day	REFERENCE	
3–4 runs per day	1.15 (0.98, 1.34)	
5+ runs per day	0.92 (0.74, 1.14)	
Rubber surface		0.005
Never competed	REFERENCE	
<6 times per year	1.01 (0.87, 1.18)	
6+ times per year	0.68 (0.53, 0.86)	
Times competed at National level		0.003
0 (never)	REFERENCE	
1	1.17 (0.96, 1.44)	
2	1.54 (1.21, 1.96)	
3	1.27 (0.93, 1.73)	
4	1.80 (1.21, 2.66)	
5	1.09 (0.68, 1.77)	
>5	1.24 (0.96, 1.60)	

Dogs who had competed at a national competition had greater odds of injury history across all number of times competing relative to those who had never competed. The dogs of handlers who reported planning around a big event (OR: 1.30) or a mix of planning around a big event and options available (OR: 1.34) had higher odds of dogs with injury history compared to handlers who reported primarily planning around trial options available. Interestingly, dogs who competed 6 or more times per year on rubber matting had a lower odds of injury history compared to dogs with no history of competing on the surface (OR: 0.68) while dogs who competed 5 or fewer times had a similar risk of injury history (OR: 1.01).

Associations with severe injury were generally smaller in magnitude for competition-level variables, and fewer variables were carried forward to adjusted model building (Table 1). Notably, the age-adjusted association between trial weekends per year and severe injury was different from that with any injury. Trialing <26+ weekends per year was associated with greater risk of severe injury, whereas the lowest risk of any injury was observed for dogs trialing the fewest weekends per year. The age-adjusted association with competing at the international level was also different for any injury (where greater risk was observed for dogs with a history of competing at the international level) and severe injury (where very little difference in risk was observed). The final adjusted model (Table 3) included only planning around big events and competing on a rubber surface, where similar associations to any injury were observed.

**Table 3:** Coefficients from adjusted model with competition level factors only for severe injury.

	**Adjusted OR (95% CI)**	**Adjusted** ***p*-value**
Dog age (per 1 year older)	1.19 (1.16, 1.23)	<0.001
Approach to competition planning		0.004
Plan around availability/schedule	REFERENCE	
Plan around a big event	1.31 (1.08, 1.60)	
Mix of the two	1.90 (1.15, 3.12)	
Other approach	1.35 (0.89, 2.03)	
Rubber surface		0.033
Never competed	REFERENCE	
<6 times per year	1.04 (0.85, 1.27)	
6+ times per year	0.65 (0.46, 0.92)	

Most training level variables were also associated with injury history in age-adjusted models (Table 4). However, in model building, only three variables remained significant at *p* < 0.05: age starting any jump training, age starting any teeter training, and age starting any weave training (Table 5). In this model, the association between injury risk and jump training was mostly in the expected direction, with younger age of starting associated with higher odds of injury except that dogs started very young (<3 months old) appeared not to be at increased risk. In adjusted models, dogs that started weave and teeter training at earlier ages (among dogs who started jump training at the same age) had lower risk of injury. Thus, the highest risk in this model belonged to dogs who started jump training early (between 3 and 12 months), but did not start training weaves or teeter before 18 months.

**Table 4:** Age adjusted associations between training risk factors and injury history.

	***N* (%)**	**Any injury** **OR (95% CI)**	**Any injury *p*-value**	**Severe injury** **OR (95% CI)**	**Severe injury *p*-value**
First started any agility-specific training			<0.001^a^		0.003^a^
<16 weeks	625 (14.9)	1.29 (1.00, 1.65)		1.00 (0.71, 1.42)	
4–6 months	876 (20.9)	1.77 (1.40, 2.23)		1.30 (0.95, 1.78)	
6–12 months	1,211 (28.9)	1.86 (1.49, 2.31)		1.63 (1.22, 2.17)	
13–18 months	657 (15.7)	1.50 (1.18, 1.91)		1.18 (0.85, 1.64)	
19–24 months	295 (7.0)	1.44 (1.07, 1.94)		1.17 (0.78, 1.75)	
2+ years	533 (12.7)	REFERENCE		REFERENCE	
Age competed in first fun match			<0.001^a^		0.020^a^
<12 months	86 (2.1)	1.04 (0.63, 1.71)		0.56 (0.23, 1.35)	
12–15 months	566 (13.6)	1.22 (0.94, 1.59)		1.12 (0.78, 1.60)	
16–18 months	951 (22.8)	1.45 (1.15, 1.84)		1.50 (1.10, 2.05)	
19–24 months	899 (21.6)	1.52 (1.20, 1.93)		1.25 (0.91, 1.72)	
25–30 months	320 (7.7)	1.44 (1.07, 1.93)		1.05 (0.70, 1.57)	
31–36 months	106 (2.5)	0.82 (0.52, 1.29)		0.85 (0.45, 1.61)	
3+ years	455 (10.9)	REFERENCE		REFERENCE	
N/A – no fun match	787 (18.9)	1.10 (0.86, 1.40)		1.00 (0.72, 1.39)	
Age competed in first trial			<0.001^a^		0.36
<16 months	203 (4.9)	0.98 (0.70, 1.38)		1.08 (0.68, 1.73)	
16–18 months	837 (20.0)	1.27 (1.03, 1.57)		1.18 (0.89, 1.56)	
19–24 months	1,566 (37.5)	1.45 (1.21, 1.75)		1.20 (0.93, 1.54)	
25–30 months	640 (15.3)	1.49 (1.19, 1.86)		1.28 (0.95, 1.73)	
31–36 months	200 (4.8)	0.99 (0.71, 1.39)		0.82 (0.50, 1.33)	
3+ years old	732 (17.5)	REFERENCE		REFERENCE	
Age any jumps			<0.001^a^		<0.001^a^
>18 months	701 (16.9)	REFERENCE		REFERENCE	
<3 months	101 (2.4)	0.66 (0.40, 1.08)		0.31 (0.11, 0.86)	
3–6 months	500 (12.1)	1.53 (1.20, 1.95)		1.02 (0.72, 1.46)	
7–9 months	852 (20.6)	1.70 (1.38, 2.11)		1.63 (1.22, 2.17)	
10–12 months	1,015 (24.5)	1.73 (1.41, 2.13)		1.49 (1.13, 1.96)	
13–15 months	744 (18.0)	1.38 (1.11, 1.72)		1.38 (1.03, 1.86)	
16–18 months	233 (5.6)	1.52 (1.11, 2.07)		1.16 (0.75, 1.79)	
Age elbow height jumps			<0.001^a^		0.009^a^
>18 months	885 (21.7)	REFERENCE		REFERENCE	
<7 months	72 (1.8)	1.06 (0.63, 1.78)		0.52 (0.20, 1.33)	
7–9 months	281 (6.9)	1.17 (0.88, 1.56)		0.95 (0.63, 1.43)	
10–12 months	897 (22.0)	1.36 (1.12, 1.66)		1.12 (0.86, 1.47)	
13–15 months	1,385 (34.0)	1.60 (1.34, 1.92)		1.43 (1.12, 1.81)	
16–18 months	553 (13.6)	1.31 (1.05, 1.64)		1.02 (0.75, 1.40)	
Age full height jumps			0.009^a^		0.35
>18 months	1,461 (35.6)	REFERENCE		REFERENCE	
<10 months	54 (1.3)	0.75 (0.41, 1.36)		0.80 (0.33, 1.93)	
10–12 months	349 (8.5)	1.00 (0.79, 1.28)		0.99 (0.70, 1.38)	
13–15 months	1,139 (27.7)	1.23 (1.05, 1.45)		1.13 (0.90, 1.41)	
16–18 months	1,104 (26.9)	1.27 (1.08, 1.50)		1.24 (0.99, 1.55)	
Age backside at any height			0.017^a^		0.75
>18 months	1,907 (50.5)	REFERENCE		REFERENCE	
<10 months	96 (2.5)	1.22 (0.80, 1.87)		0.96 (0.51, 1.79)	
10–12 months	354 (9.4)	1.18 (0.93, 1.51)		1.08 (0.76, 1.53)	
13–15 months	701 (18.6)	1.31 (1.09, 1.58)		1.09 (0.84, 1.42)	
16–18 months	718 (19.0)	1.28 (1.07, 1.53)		1.18 (0.92, 1.52)	
Age backside at full height			0.013^a^		0.67
>18 months	2,246 (58.9)	REFERENCE		REFERENCE	
<13 months	176 (4.6)	0.90 (0.64, 1.25)		0.74 (0.44, 1.23)	
13–15 months	579 (15.2)	1.30 (1.07, 1.58)		1.03 (0.78, 1.36)	
16–18 months	810 (21.3)	1.20 (1.01, 1.42)		1.02 (0.80, 1.29)	
Tunnel age			<0.001^a^		<0.001^a^
>18 months	641 (15.5)	REFERENCE		REFERENCE	
<3 months	600 (14.5)	1.14 (0.90, 1.46)		0.74 (0.51, 1.08)	
3–6 months	1,080 (26.2)	1.60 (1.30, 1.97)		1.45 (1.09, 1.92)	
7–9 months	713 (17.3)	1.70 (1.36, 2.14)		1.67 (1.23, 2.26)	
10–12 months	513 (12.4)	1.56 (1.23, 1.99)		1.56 (1.12, 2.15)	
13–15 months	396 (9.6)	1.51 (1.16, 1.96)		1.44 (1.02, 2.04)	
16–18 months	184 (4.5)	1.43 (1.01, 2.01)		1.13 (0.69, 1.85)	
Curved tunnel age			<0.001^a^		0.001^a^
>18 months	705 (17.0)	REFERENCE		REFERENCE	
<3 months	208 (5.0)	1.10 (0.79, 1.54)		0.57 (0.32, 1.01)	
3–6 months	857 (20.6)	1.40 (1.14, 1.73)		1.11 (0.82, 1.49)	
7–9 months	935 (22.5)	1.48 (1.20, 1.82)		1.48 (1.12, 1.95)	
10–12 months	684 (16.5)	1.59 (1.27, 1.99)		1.48 (1.10, 2.00)	
13–15 months	506 (12.2)	1.54 (1.22, 1.96)		1.50 (1.09, 2.05)	
16–18 months	259 (6.2)	1.22 (0.91, 1.65)		0.96 (0.62, 1.48)	
Aframe age			0.003^a^		0.18^a^
>18 months	1,159 (28.1)	REFERENCE		REFERENCE	
<10 months	317 (7.7)	1.13 (0.87, 1.46)		0.85 (0.58, 1.24)	
10–12 months	821 (19.9)	1.02 (0.84, 1.23)		1.05 (0.81, 1.35)	
13–15 months	1,203 (29.2)	1.35 (1.14, 1.60)		1.16 (0.92, 1.46)	
16–18 months	626 (15.2)	1.27 (1.03, 1.55)		1.30 (0.99, 1.70)	
Dogwalk age			0.002^a^		0.31
>18 months	1,150 (27.9)	REFERENCE		REFERENCE	
<10 months	423 (10.3)	0.90 (0.71, 1.14)		0.83 (0.59, 1.16)	
10–12 months	934 (22.6)	1.16 (0.97, 1.39)		1.00 (0.78, 1.28)	
13–15 months	1,083 (26.2)	1.35 (1.14, 1.61)		1.17 (0.93, 1.48)	
16–18 months	537 (13.0)	1.12 (0.90, 1.39)		1.07 (0.80, 1.44)	
Teeter age			0.002^a^		0.50
>18 months	1,134 (28.9)	REFERENCE		REFERENCE	
<10 months	389 (9.9)	0.80 (0.63, 1.02)		0.83 (0.58, 1.17)	
10–12 months	847 (21.6)	1.11 (0.92, 1.33)		1.02 (0.79, 1.31)	
13–15 months	1,010 (25.8)	1.26 (1.06, 1.50)		1.13 (0.89, 1.43)	
16–18 months	543 (13.8)	1.23 (0.99, 1.52)		1.08 (0.81, 1.44)	
Any weaves age			0.005^a^		0.129^a^
>18 months	1,037 (24.8)	REFERENCE		REFERENCE	
<7 months	205 (4.9)	1.10 (0.80, 1.50)		0.81 (0.50, 1.31)	
7–9 months	462 (11.1)	1.06 (0.84, 1.33)		1.02 (0.74, 1.41)	
10–12 months	825 (19.7)	1.04 (0.86, 1.26)		0.99 (0.76, 1.30)	
13–15 months	1,148 (27.5)	1.37 (1.15, 1.64)		1.21 (0.95, 1.54)	
16–18 months	503 (12.0)	1.25 (1.00, 1.56)		1.35 (1.01, 1.81)	
Sequences closed weaves			0.002^a^		0.001^a^
>18 months	1,464 (35.0)	REFERENCE		REFERENCE	
<10 months	135 (3.2)	0.74 (0.50, 1.08)		0.47 (0.23, 0.94)	
10–12 months	495 (11.8)	1.00 (0.81, 1.24)		1.12 (0.83, 1.49)	
13–15 months	1,250 (29.9)	1.22 (1.04, 1.42)		1.10 (0.88, 1.38)	
16–18 months	838 (20.0)	1.31 (1.10, 1.56)		1.50 (1.18, 1.89)	
Aframe contact			0.77		0.60
2 on 2 off	1,900 (47.7)	REFERENCE		REFERENCE	
4 on	133 (3.3)	0.86 (0.59, 1.25)		0.86 (0.59, 1.25)	
Other/no specific behavior	164 (4.1)	1.09 (0.78, 1.51)		1.09 (0.78, 1.51)	
Running	1,785 (44.8)	0.97 (0.85, 1.11)		0.97 (0.85, 1.11)	
Dogwalk contact			0.023^a^		0.12^a^
2 on 2 off	2,528 (60.3)	REFERENCE		REFERENCE	
4 on	199 (4.8)	0.78 (0.58, 1.06)		0.74 (0.48, 1.15)	
Other/no specific behavior	146 (3.5)	0.99 (0.71, 1.40)		0.90 (0.56, 1.43)	
Running	1,317 (31.4)	0.82 (0.71, 0.94)		0.80 (0.66, 0.98)	
Teeter contact			<0.001^a^		0.028^a^
2 on 2 off	2,203 (52.7)	REFERENCE		REFERENCE	
4 on (down)	311 (7.4)	0.73 (0.56, 0.93)		0.76 (0.52, 1.09)	
4 on (standing)	1,200 (28.7)	0.78 (0.67, 0.90)		0.87 (0.72, 1.07)	
No specific behavior	240 (5.7)	0.69 (0.52, 0.92)		0.65 (0.43, 0.98)	
Other	224 (5.4)	0.74 (0.55, 0.99)		0.58 (0.37, 0.91)	
Weave training method			0.14^a^		<0.001^a^
2 ×2	1,912 (45.8)	REFERENCE		REFERENCE	
Channel	1,329 (31.8)	0.86 (0.74, 0.99)		0.67 (0.54, 0.82)	
Guide wires	462 (11.1)	0.99 (0.80, 1.22)		0.68 (0.51, 0.92)	
Other	474 (11.4)	0.86 (0.70, 1.06)		0.66 (0.49, 0.89)	

a *p < 0.20 and retained for model building*.

**Table 5:** Coefficients from adjusted model with training level factors for any injury.

	**Adjusted OR**	**Adjusted**
	**(95% CI)**	***p*-value**
Dog age (per 1 year older)	1.17 (1.14, 1.20)	<0.001
Age any jumps		<0.001
>18 months	REFERENCE	
<3 months	0.98 (0.54, 1.75)	
3–6 months	2.39 (1.66, 3.43)	
7–9 months	2.47 (1.77, 3.44)	
10–12 months	2.24 (1.63, 3.07)	
13–15 months	1.58 (1.16, 2.15)	
16–18 months	1.55 (1.09, 2.20)	
Teeter age		0.018
>18 months	REFERENCE	
<10 months	0.59 (0.43, 0.83)	
10–12 months	0.82 (0.62, 1.08)	
13–15 months	0.92 (0.71, 1.19)	
16–18 months	0.94 (0.73, 1.23)	
Any weaves age		0.034
>18 months	REFERENCE	
<7 months	0.89 (0.58, 1.37)	
7–9 months	0.75 (0.53, 1.07)	
10–12 months	0.67 (0.49, 0.92)	
13–15 months	0.92 (0.69, 1.24)	
16–18 months	0.89 (0.66, 1.20)	

Associations with severe injury were generally similar for training level factors (Table 4). Notably, the weave training method showed greater association with severe injury (more severe injury among those trained with 2 ×2 method than all others). In the final adjusted model for severe injury three variables were significant at *p* < 0.05: age starting curved tunnels, age sequencing with closed weave poles, and weave training method (Table 6). In this adjusted model, all methods for weave training showed lower odds of severe injury compared to the 2 ×2 method, and the lowest risk of severe injury was observed among dogs who started curved tunnels and sequencing closed weaves at the youngest ages. A general increase in risk was observed for starting curved tunnels between 3 and 15 months, holding the weave factors constant.

**Table 6:** Coefficients from adjusted model with training level factors for severe injury.

	**Adjusted OR**	**Adjusted**
	**(95% CI)**	***p*-value**
Dog age (per 1 year older)	1.20 (1.17, 1.24)	<0.001
Curved tunnel age		0.001
>18 months	REFERENCE	
<3 months	0.74 (0.48, 1.14)	
3–6 months	1.39 (0.97, 1.98)	
7–9 months	1.55 (1.08, 2.24)	
10–12 months	1.40 (0.96, 2.04)	
13–15 months	1.31 (0.90, 1.91)	
16–18 months	1.05 (0.64, 1.75)	
Sequences closed weaves		0.037
>18 months	REFERENCE	
<10 months	0.50 (0.24, 1.04)	
10–12 months	1.08 (0.76, 1.53)	
13–15 months	0.97 (0.73, 1.28)	
16–18 months	1.29 (0.97, 1.70)	
Weave training method		0.002
2 ×2	REFERENCE	
Channel	0.69 (0.56, 0.85)	
Guide wires	0.73 (0.54, 0.99)	
Other	0.72 (0.53, 0.97)	

## Discussion

As was hypothesized, starting jump training at an earlier age was associated with greater risk of injury relative to starting after 18 months. The lower risk of injury associated with dogs starting jump training at over 18 months of age could be due to the fact that the majority of dogs are skeletally mature at 18 months. It is possible that the high impact of jump training before skeletal maturity may increase the risk of injuries or musculoskeletal conditions. High impact repetitive sport activities in children have been shown to contribute to primary periphyseal stress injuries (13, 14). Previous canine biomechanical studies evaluating the effects of jumping on forelimb muscular activation have shown that the jump task is the most physiologically demanding task for all evaluated forelimb muscles (15). A study by Söhnel et al. evaluated limb length and stiffness during jumping in agility dogs with greater than and less than 4 years of agility experience (16). They found that, during landing, beginner dogs (those with <4 years of agility experience) had 17% higher limb compression during stance phase (16). No studies have evaluated how this higher limb compression and higher muscular activation may impact development of bones and joints in skeletally immature dogs.

It is common practice to start younger dogs jumping lower jump heights, and to increase the jump height as the dog ages and approaches skeletal maturity, often determined by radiographic closure of the growth plates. Jumping a lower jump height is thought to exert less force on the developing bones and joints, and therefore be less likely to cause developmental musculoskeletal conditions or injury. While biomechanical studies have shown that increasing jump height increases peak vertical force upon landing with the forelimbs, and increases angulation of the scapulohumeral and sacroiliac joints, no studies have correlated the kinematic and kinetic findings with injury development or risk (17, 18). Based on the findings of this survey, it does not appear that starting jumping at a lower jump height when younger is protective of injury. However, competition jump height in relation to dog height was correlated with injury risk. Jumping 2–4” and jumping >4” above the height of the withers was associated with an increased risk of injury. This finding may be due to the increased neck angulation, lumbar spine extension and shoulder flexion as jump height increases, as well as the increased peak vertical force with higher jump heights and steeper landing angles (18, 19). Further studies are needed to prospectively evaluate effect of jump training on musculoskeletal development and injury incidence, as well as the association of altered kinetics and kinematics of increasing jump height and injury development.

Many variables thought to be associated with injury risk in agility dogs were not significant in adjusted models. Counter to our hypothesis, contact method (stopped vs. running) was not predictive in adjusted models. We hypothesized that stopped contacts would be correlated with a higher injury risk due to the increased deceleration (braking) forces experienced during downhill locomotion (20). Multiple studies have evaluated the kinetics and kinematics of the A-frame obstacle (15, 21–23). These studies have shown that ascent up a full height A-frame requires greater propulsive forces than a lower height A-frame (22), that range of motion in the lumbar spine changes during the different phases of obstacle completion, with lumbar flexion noted during the section of incline to apex and lumbar extension noted during the approach to incline and again from the apex to decline sections (23). As injury is likely multi-factorial, there are conflicting variables that make it hard to elucidate the exact effects of particular variables on injury risk. Training running contacts instead of stopped contacts, especially a running dogwalk, has become more popular in recent years as a way to increase course speed and competitiveness. Other data from this survey indicates that there may be a link between competitiveness (higher levels of competition, participation in national/international events) and injury risk. Therefore, it is possible that running contacts, in and of themselves, are associated with reduced mechanical loads and decreased injury risk, but this benefit is counteracted by their more common use among faster, highly competitive dogs.

Anecdotally it is thought that performing the weave obstacle places substantial stress on the shoulders and spine and that, as a result, training weaves before skeletal maturity is not recommended. In the adjusted model, there was a decreased risk of injury when weave training was started prior to 7 months of age (and all ages prior to 18 months), leading us to reject this hypothesis. It is unknown whether this represents a true decrease in injury risk since some combinations of starting ages were observed only in a small number of dogs (e.g., most dogs started jump training prior to weaves). If starting weave training early does reduce injury risk it is possible that weave training improves overall body awareness and coordination, which has been shown to decrease injury risk in human athletes (24–26). Agility, balance and coordination training is recommended in pre-pubescent human athletes in order to take advantage of increased synaptoplasticity and prevent injuries (27). Weave training method, while not retained in the model for general injury risk, was retained in the model for severe injury risk. Dogs who did the 2 ×2 weave training method had an increased risk of severe injury compared to the channel method or guide-wire method of training. It is possible that the 2 ×2 method requires more repetitions during training, thereby resulting in overuse or overtraining injuries. Biomechanical studies are needed to evaluate kinetics and kinematics of weave obstacle execution and different training methods, and how that may relate to injury risk and prevention.

Surprisingly, early age at completing a final teeter behavior was also associated with decreased risk of injury in the adjusted model. This observation could be due to more experienced or more effective trainers/handlers teaching these dogs, thereby being able to complete this particular training earlier. Teeter training also involves more balance and coordination than other contact behaviors. It is possible that the dogs that are able to learn this behavior quickly and early have more coordination and body awareness than dogs that take longer to learn this skill, thereby possibly decreasing the risk of injury. In human studies, improved balance is correlated with decreased injury risk and enhanced athletic performance (28). While there are no studies evaluating the effect of balance on canine injury risk or athletic performance, there are likely to be similarities to the effects found in the human literature.

In the Cullen et al. study, 26.8% of injuries reported had an undefined or non-specific cause of injury, i.e., the injury was not caused by contact with a certain obstacle or in relation to surface type (29). This subset of injuries may be due to chronic overuse or overtraining. We had hypothesized that increasing number of competition days per year and runs per competition day would be associated with increased injury risk. Increased competition load, defined as the cumulative amount of stress placed on an individual from single or multiple competitions over a period of time (30), has been associated with increased injury risk in the human literature (31). Based on the data from this survey there was no association between frequency of competition days per year and injury risk. There was, however, an association between number of runs per competition day and injury risk. Runs per day was associated with injury, with 3-4 runs per day having an increased risk compared to 1–2 runs per day. Injury risk is increased if the intensity, frequency or duration of loading is beyond the tissue's capacity or if the recovery between loading is insufficient (30). It is possible that 3–4 runs a day increases injury risk due to decreased recovery or tissue overload, compared to 1–2 runs a day. It has also been suggested that fatigue due to repetitive loading may increase the susceptibility to injury (30), which could also play a role in the increased injury rates in the 3–4 runs per day (32). The reported decreased injury risk in dogs who complete 5+ runs per day is likely reverse causality, as dogs who have sustained an injury are less likely to be capable of 5+ runs a day or the handlers are more cautious about the number of runs. These data indicate a need for more studies evaluating competition load in our canine athletes and how that load affects injury development.

Periodization is the process of planning training programs to include variations in training loads and cycles in order to maximize physiological adaptations for competition performance (33). The human literature evaluating periodization techniques is extensive and complex. There are many periodization methods and the training and competition needs vary by sport. Human studies have shown that detailed training scheduling and periodization results in improved strength and decreased risk of sports related injuries, but this has not been evaluated in canine athletes (24–26, 30, 33–38). There is also evidence in the human literature that training periodization lowers the risk of overtraining and increases the chance of peaking at key competitions (33). Periodization is not currently a consideration for the majority of canine athletes and there are no studies evaluating the effect periodization has on canine, or even equine, athletic performance.

In evaluating periodization in this study, contrary to expectation, we found that canine athletes whose owners planned training and competition schedules actually had a higher risk of injury. This is likely due to multiple factors. This survey was retrospective in nature, and exact training and planning methods could not be assessed. It is also possible that owners were not performing true periodization with cycles of very specific increases and decreases in training load, but instead more calendar schedule planning of training days and competition days. The limitations of the retrospective survey combined with the complexity of periodization techniques makes it challenging to assess relationship to injury. However, the relationship between injury risk and training planning could be correlated with the hypothesis that faster, more competitive dogs are more likely to get injured, as the owners of these dogs are more likely to be planning their competitions and training based on large national and international events. Prospective studies are also needed to evaluate the effect of true training periodization on canine athletic performance and injury risk.

Injury risk in relation to competition and training surfaces has been extensively evaluated in equine and human medicine (3, 39, 40). Relationships between surface type and risk of injury have been minimally evaluated in canine sports, with one study suggesting a correlation between track surface and injury in racing greyhounds (41). We had originally hypothesized that competing on a turf surface would have the lowest risk of injury. Surprisingly, and counter to our hypothesis, dogs that competed on rubber matting had a lower risk of injury. Rubber matting has fallen out of favor in many agility venues due to the thought that there is an increased risk of slipping on that particular surface. It is possible that handlers with faster dogs specifically choose to not compete in venues with rubber matting due to concern for injury, which would also support the correlation of speed with injury risk. There may be other factors involved with the dogs that are competing more frequently on rubber that potentially decreases their injury risk, confounding the correlation between rubber matting and injury risk. More studies are needed to prospectively evaluate speed and correlation with injury in agility dogs, as well as evaluate the effect that surface has on biomechanics, performance and injury risk.

Limitations of this study include those associated with a cross-sectional, retrospective survey. These include potential self-selection bias which may result in the survey sample not being representative of the total agility dog population. Participant recall and handler-reported data may have also resulted in potential inaccuracies. Also, since this survey was in English but distributed world-wide, it is possible that there were inaccuracies due to variations in terminology and training methods between countries and geographical regions. Future studies should consider collaborating with agility organizations to obtain data from all competitors in order to address potential sampling error and self-selection bias.

These data provide valuable current insight into the possible effects that training and competition variables may have on injury risk in agility dogs. While no definitive recommendations can be made regarding training or competition based on these data, they provide a starting point for future, prospective studies. Specifically, this survey indicates a need for further studies evaluating the biomechanics of agility obstacles and obstacle training techniques and their effect on musculoskeletal development and injuries. There is also a significant need for studies evaluating strength and conditioning programs and training periodization in canine athletics, both for performance and injury prevention. With the increasing popularity of companion dog sports, there is a definitive need for research on sport specific training and injury prevention in order to provide better training and care recommendations to these canine athletes.

## Data Availability Statement

The raw data supporting the conclusions of this article will be made available by the authors, without undue reservation.

## Ethics Statement

Ethical review and approval was not required for the animal study because the Ohio State University Office of Responsible Research Practices determined the project was exempt from IRB review because it was an owner-based internet survey and the information was recorded without direct or indirect identifiers. The patients/participants provided their written informed consent to participate in this study.

## Author Contributions

APM, AS, and NK contributed to the study idea, survey, and study design. AS collected data, performed data entry, and statistical analysis. All authors contributed to the manuscript preparation and revisions for submission.

## Conflict of Interest

The authors declare that the research was conducted in the absence of any commercial or financial relationships that could be construed as a potential conflict of interest.

## Publisher's Note

All claims expressed in this article are solely those of the authors and do not necessarily represent those of their affiliated organizations, or those of the publisher, the editors and the reviewers. Any product that may be evaluated in this article, or claim that may be made by its manufacturer, is not guaranteed or endorsed by the publisher.

## Footnotes

^1^Personal communication. Carrie DeYoung, Director of AKC Agility. June 30, 2020.

^2^Qualtrics survey software, Provo UT.

^3^Copies of the questionnaire are available from the corresponding author on request.

^4^Facebook.
